# Fibroblastic growth factor receptor 1 amplification in osteosarcoma is associated with poor response to neo-adjuvant chemotherapy

**DOI:** 10.1002/cam4.268

**Published:** 2014-05-27

**Authors:** M Fernanda Amary, Hongtao Ye, Fitim Berisha, Bhavisha Khatri, Georgina Forbes, Katie Lehovsky, Anna M Frezza, Sam Behjati, Patrick Tarpey, Nischalan Pillay, Peter J Campbell, Roberto Tirabosco, Nadège Presneau, Sandra J Strauss, Adrienne M Flanagan

**Affiliations:** 1 Histopathology, London Sarcoma Service, Royal National Orthopaedic Hospital NHS TrustStanmore, Middlesex, HA7 4LP, U.K; 2 UCL Cancer InstituteHuntley Street, London, WC1E 6BT, U.K; 3 University College London Hospitals Foundation Trust, London Sarcoma Service250 Euston Road, London, NW1 2PG, U.K; 4 Cancer Genome Project, Wellcome Trust Sanger InstituteWellcome Trust Genome Campus, Hinxton, Cambridgeshire, CB10 1SA, U.K

**Keywords:** Amplification, FGFR, *FGFR1*, FISH, genetics, osteosarcoma, polysomy

## Abstract

Osteosarcoma, the most common primary bone sarcoma, is a genetically complex disease with no widely accepted biomarker to allow stratification of patients for treatment. After a recent report of one osteosarcoma cell line and one tumor exhibiting *fibroblastic growth factor receptor 1* (*FGFR1*) gene amplification, the aim of this work was to assess the frequency of *FGFR1* amplification in a larger cohort of osteosarcoma and to determine if this biomarker could be used for stratification of patients for treatment. About 352 osteosarcoma samples from 288 patients were analyzed for *FGFR1* amplification by interphase fluorescence in situ hybridization. *FGFR1* amplification was detected in 18.5% of patients whose tumors revealed a poor response to chemotherapy, and no patients whose tumors responded well to therapy harbored this genetic alteration. *FGFR1* amplification is present disproportionately in the rarer histological variants of osteosarcoma. This study provides a rationale for inclusion of patients with osteosarcoma in clinical trials using *FGFR* kinase inhibitors.

## Introduction

Osteosarcoma is the most common primary sarcoma of bone, occurring rarely before the age of 4 years, and seen most commonly in adolescents with a second peak in adults, over 40 years of age. The vast majority of osteosarcomas represents high-grade disease and behave in an aggressive manner. The 5-year survival for patients with appendicular high-grade disease who present without metastases is ∼60–70%, whereas prior to the introduction of neo-adjuvant chemotherapy only 20% of patients survived this period 1. Histologically it is a heterogeneous disease, with marked inter and intratumor variation 2. It is also a genetically complex disease, with a high burden of karyotypic abnormalities and a high level of genomic instability 3. Apart from the recurrent amplification of *MDM2*, which is found in the majority of parosteal osteosarcomas, for the most part a low-grade disease, and in low-grade central osteosarcomas, there are no other widely accepted biological markers which subclassify the histological variants of osteosarcoma 2,4.

Neo-adjuvant chemotherapy is the conventional treatment for all histological variants of high-grade osteosarcoma and response to such therapy is one of the most powerful predictors of outcome in patients with resectable disease 5: a “good response” is classified as a tumor showing 90% or more necrosis and ‘poor’ if there is less than 90% tumor necrosis in response to chemotherapy. Although there are several reports correlating gene expression with clinical outcome, which are useful in terms of providing insight into the biology of the disease, none are used in a clinical setting to determine clinical management 6–11. Indeed, there are no widely accepted biomarkers employed for predicting response to therapy that allow stratification of patients for treatment type 12.

Recently, it was reported that one of 7 osteosarcoma cell lines, and one of 17 osteosarcomas harbored amplification of *fibroblastic growth factor receptor 1* (*FGFR1*), a receptor tyrosine kinase located on chromosome 8p12, which leads to activation of the Ras/mitogen-activated protein kinase and PI3/Akt pathway, and ultimately leading to cell proliferation and differentiation 13. The biological significance of this genetic alteration was supported by inhibition of cell growth in the osteosarcoma cell line, G292, harboring the *FGFR1* amplification, by the FGFR inhibitor, NVP-BGJ398, and was reinforced by cell growth suppression following silencing of *FGFR1* using shRNAs 13. This report prompted us to assess the frequency of *FGFR1* amplification in a larger cohort of osteosarcomas, with the aim of determining if this biomarker could be used for identifying a histological subtype/s of osteosarcoma that may benefit from treatment with FGFR1 inhibitors, and also to determine if *FGFR1* amplification would allow stratification of patients for treatment with neo-adjuvant therapy and/or introduction of specific FGFR inhibitors as a treatment option.

## Materials and Methods

### Ethical approval and samples

The samples were obtained from the Stanmore Musculoskeletal Biobank, approved by the Cambridgeshire Research Ethics committee, Cambs., U.K.: Reference Number: 09/H0304/78).

Tumor samples were retrieved through searching the RNOH NHS Trust electronic histopathology database between 2000 and 2012. The diagnoses were reviewed and subtyped using the WHO classification (AMF, RT, MFA) 2 and sections were selected for assessment of *FGFR1* amplification. Tissue microarrays (TMAs) were constructed as previously reported using a manual tissue arrayer (Beecher Instruments Inc, Sun Prairie, WI) using at least two representative 1 mm cores of tumor 14. All tumors classified as “good responders” to chemotherapy were analyzed on the pretreatment biopsy specimen. All amplification-positive cases were analyzed in more than one sample (pre and post –treatment, and recurrent disease) where tissue was available.

### Fluorescence *in situ* hybridisation

FISH was performed using the commercially available ZytoLight *SPEC FGFR1/Centromere (CEN)8* Dual Color Probe (ZytoVision, Bremerhaven, Germany). FGFR1 probes are labeled green and CEN8 orange. FISH was performed as previously described. In brief, deparaffinised sections were pretreated with deionized water in a pressure cooker for 5 min and digested with pepsin at 37°C for 50 min. Subsequently, the tissue sections and FGFR1/CEN8 FISH probe were codenatured at 72°C for 10 min and hybridized overnight at 37°C. Following hybridization, washing was performed. Slides were then counterstained with 4′, 6-diamidino-2-phenylindole (DAPI) and mounted with coverslips.

At least 50 nonoverlapping nuclei were scored for the number of *FGFR1* and CEN8 copies at 100× oil immersion objective, after initial scanning of the section using a 40× objective to detect areas showing copy number variation after which areas with the highest copy number were counted using a fluorescence microscope (Olympus BX61, Southend-on-Sea, U.K.) equipped with appropriate filters, a charge-coupled device camera (Olympus XM10), and the FISH imaging and capturing software Cell* Imaging system (Olympus Soft Imaging Solution, Germany).

Amplification was classified as positive if ≥10% of the cells showed (a) *FGFR1*/CEN8 ratio >2, (b) clusters of *FGFR1* signals, or (c) >15 copies of *FGFR1* per cell 15. Tumors with polysomy of chromosome 8 comprised two categories: high-level polysomy (≥4 copies of the gene of interest and CEN8/cell in ≥40% of cells), and low-level polysomy of chromosome 8 (>2 copies of the gene of interest and CEN8/cell in ≤40% of cells, and 3 copies of the gene of interest per cell in ≥40% of cells). Disomy was defined as two copies of the gene of interest and CEN8 in >90% of the cells.

The FISH slides were assessed by HY and FB independently. If there were a discrepancy, the slides were reviewed by MFA and AMF and a consensus was reached. If a result was equivocal, the FISH was repeated on a full tissue section. All equivocal cases were reviewed by MFA and AMF.

### Patient characteristics and outcome analysis

Clinical details including age, sex, site of primary tumor, and presence and absence of metastases were collated from pathology and patient records where available. A retrospective outcome analysis was performed on patients with extremity tumors who were known to have received chemotherapy and where follow-up information was available. The analysis was therefore not of consecutive patients treated within the London Sarcoma Service (LSS), and included patients treated outside the service. Patients received standard chemotherapy regimens according to local and clinical trial protocols in use at the time of diagnosis. This incorporated cisplatin and doxorubicin in older patients (over 40 years) or those treated in the early 2000s. The majority of patients received MAP (methotrexate, doxorubicin, and cisplatin). Overall survival (OS) was calculated as the period from diagnosis to death or last follow-up. Descriptive analysis was made using median values and range. Survival analysis was performed by Kaplan–Meier product-limit method and the differences in term of OS according to pathological response were evaluated by the log-rank test. SPSS software (version 17.00, SPSS, Chicago, IL) was used for statistical analysis. A *P* value of less than 0.05 was considered to indicate statistical significance.

## Results

To investigate *FGFR1* amplification in osteosarcoma, we evaluated a total of 352 samples from 288 patients. About 275 with osteosarcomas arising in bone and 13 arising in soft tissue gave informative results for FGFR1/CEN8 FISH. The cohort of patients with bone tumors included 119 “poor responders” and 80 “good responders” to neo-adjuvant chemotherapy. The remaining patients (*n* = 76) had either not been exposed to neo-adjuvant therapy (*n* = 57) or the response to therapy was not available (*n* = 19) (Table1).

**Table 1:** Correlation of data from 288 patients including osteosarcoma histological phenotype, response to neo-adjuvant chemotherapy, and the presence and absence of *FGFR1* amplification.

Osteosarcoma details	Histological subtype (*n*)	*FGFR1* amplification negative	*FGFR1* amplification positive
Good response^2^ (*N* = 80)	Osteoblastic (48)	48	0
	Chondroblastic (19)	19	0
	Fibroblastic/Pleomorphic (5)	5	0
	Telangiectatic (6)	6	0
	Periosteal (1)	1	0
	High-grade surface (1)	1	0
	Total	80	0
Poor response^2^ (*N* = 119)	Osteoblastic (61)	54	7
	Chondroblastic (24)	20	4
	Fibroblastic/Pleomorphic (18)	10	8
	Telangiectatic (8)	6	2
	Rare subtypes^1^ (4)	4	0
	Periosteal (3)	2	1
	Parosteal (1)	1	0
	Total	97	22
Soft tissue osteosarcoma (*N* = 13)	Soft tissue osteosarcoma	11	2
NCG or unknown^2^ (*N* = 76)	Parosteal (15)	15	0
	Periosteal (4)	4	0
	High-grade surface (1)	1	0
	Osteoblastic (18)	18	0
	Chondroblastic (6)	5	1
	Fibroblastic/Pleomorphic (13)	12	1
	Telangiectatic (6)	6	0
	Low-grade central (7)	7	0
	Rare subtypes^1^ (6)	6	0
	Total	74	2

The shaded area highlights surface osteosarcomas.NCG, No chemotherapy given.

1 Osteoblastoma-like, giant-cell rich, fibrous-dysplasia-like.

2 Bone osteosarcomas.

**Figure 1:**
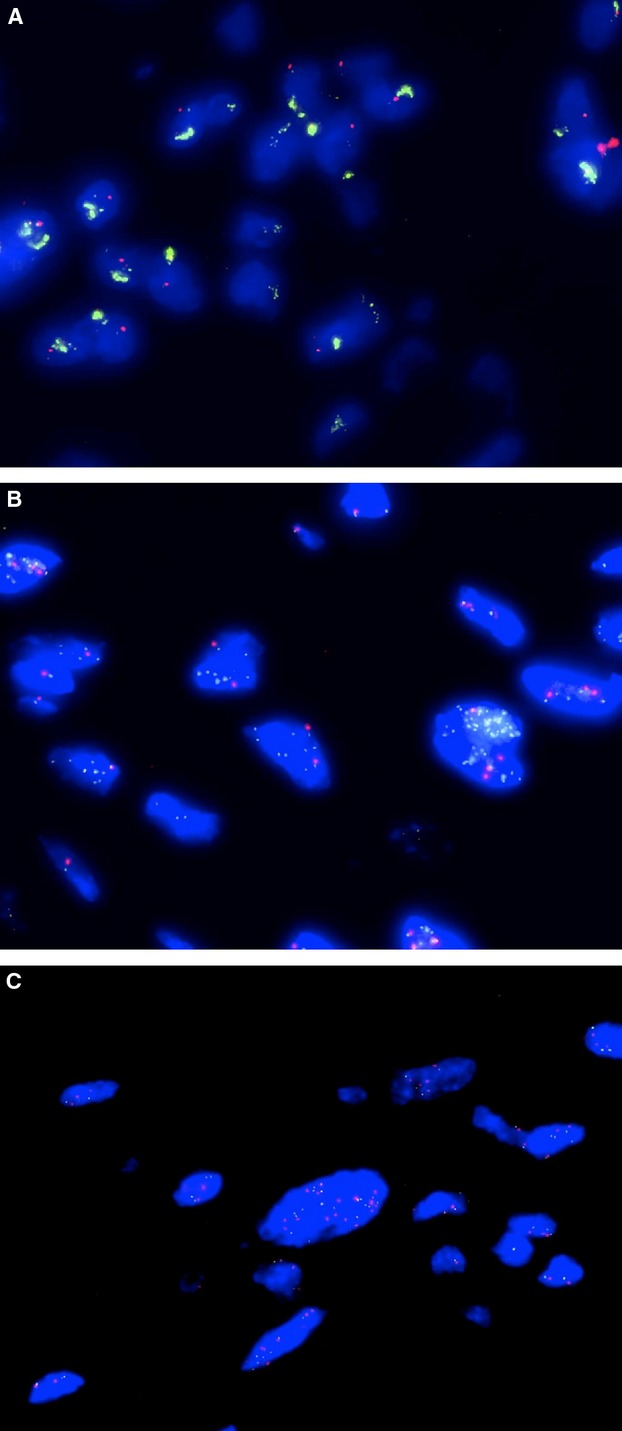
Photomicrographs of FISH for *FGFR1*/CEN8 showing clusters of *FGFR1* signals (A), *FGFR*/CEN8 ratio >2 (B) and >15 copies of *FGFR* (C).

*FGFR1* gene amplification was detected in 24 (9.6%) of 275 osteosarcomas arising in bone (Fig.1). This included 22 (18.5%) of 119 “poor responders”, whereas no tumor which revealed a “good response” to neo-adjuvant chemotherapy (*n* = 109) exhibited *FGFR1* amplification (Table2). In four of these 22 cases, samples from metastatic disease were available which also revealed *FGFR1* amplification.

**Table 2:** Morphological subtypes of classic variants of primary central high-grade osteosarcomas treated with neo-adjuvant chemotherapy: correlation with response to neo-adjuvant chemotherapy and presence of *FGFR1* amplification.

Osteosarcoma subtype	Osteoblastic 105 (57.1%)	Chondroblastic 40 (21.7%)	Fibroblastic 21 (11.4%)	Telangiectatic 14 (7.6%)	Others 4 (2.2%)	Total 184 (100%)
Number of “Good responders” (%)	45	19	5	6	0	75 (40.8)
Number of ‘Poor responders’ (%)	60	21	16	8	4	109 (59.2)
Number of cases with amplification (% of subtype)^1^	7 (6.7)	3 (7.5)	7 (33.3)	2 (14.3)	0	19

1 All cases with *FGFR1* amplification showed a poor response to neo-adjuvant chemotherapy.

One of 57 (1.7%) bone osteosarcomas not treated with neo-adjuvant chemotherapy, and one radiation-induced osteosarcoma of 19 (5.3%) bone osteosarcomas on which treatment information was not available also revealed *FGFR1* amplification.

Of the total cohort of 288 patients, 22 had a radiation-induced bone osteosarcoma, 3 (13.6%) of which showed *FGFR1* gene amplification. In none of these cases was the original tumor material available to assess for the presence of *FGFR1* amplification. Seven (31.8%) of the radiation-induced osteosarcomas revealed *c-MYC* amplification (Table3). Also included in the study were 26 surface osteosarcomas (Table1), 20 of which had not received neo-adjuvant chemotherapy. *FGFR1* amplification was detected in only one of these 26 cases (6.8%), and this tumor was a periosteal osteosarcoma. No low-grade central osteosarcoma and no Pagetic osteosarcomas exhibited *FGFR1* gene amplification.

**Table 3:** Details of radiation-induced osteosarcoma.

Neo-adjuvant treatment status	Gender	Age at diagnosis	Site	Subtype	Primary irradiated tumor; year of treatment	*FGFR1* FISH copy number	*c-MYC* copy number
MGORSP	M	46	Femur	Osteoblastic	Fibrosarcoma; 1968	DI	DI
MPORSP	M	52	Pelvis	Fibroblastic	Giant-cell tumor of bone; Right Pubis; 1980	AMP	Poly (H)
MPORSP	F	69	Pelvis	Chondroblastic	Carcinoma; cervix; 1985	DI	Poly (H)
NK	M	15	Skull	Osteoblastic	Medulloblastoma; posterior fossa; 1997	Poly (L)	AMP
MPORSP	F	26	Scapula	Chondroblastic	Rhabdomysosarcoma; chest wall; 1984	AMP	AMP
NK	F	63	Ilium	Chondroblastic	Squamous cell carcinoma; cervix; 1987	AMP	Poly (L)
MPORSP	M	34	Femur	Chondroblastic	Ewing sarcoma; femur; 1998	Poly (H)	AMP
MPORSP	F	70	Pelvis	Fibroblastic	Squamous cell carcinoma; anal canal; 1993	Poly (H)	AMP
NK	M	56	Mandible	Osteoblastic	Bilateral acinic cell carcinoma; parotid glands; 2005	Poly (L)	Poly (H)
NK	M	33	Iliac crest	Osteoblastic	Hodgkin's lymphoma; 1992	DI	Poly (L)
NK	F	38	Sacrum	Osteoblastic	Carcinoma; cervix; 2006	DI	DI
NCG	F	70	Humerus	Osteoclast-rich	Carcinoma; ductal; 1990	Poly (H)	Poly (H)
MPORSP	M	15	Mandible	Osteoblastic	Rhabdomyosarcoma; temporalis; 1995	Poly (L)	Poly (L)
NK	F	60	Vertebra	Osteoblastic	Myeloma; unknown	DI	AMP
NK	M	68	Skull	Fibroblastic	Squamous cell Carcinoma; maxilla; 2000	Poly (L)	Poly (L)
MGORSP	M	19	Mandible	Osteoblastic	Rhabdomyosarcoma; ethmoid; 1991	DI	DI
NK	F	63	Chest wall	Osteoblastic	Carcinoma; brest; 2002	Poly (H)	Poly (H)
NK	M	46	Mandible	Fibroblastic	Squamous cell carcinoma; mouth; 2003	Poly (H)	Poly (L)
NK	M	46	Sacrum	Chondroblastic	Carcinoma; rectal; 1997	Poly (L)	Poly (L)
NK	M	50	Mandible	Chondroblastic	Squamous cell carcinoma; tongue; 1990	Poly (H)	Poly (H)
NK	M	72	Skull	Telangiectatic	Bilateral retinoblastoma; 1935	DI	DI
NK	F	48	Chest wall	Chondroblastic	Carcinoma; breast; 1987	DI	AMP

Poor response, MGORSP. NCG, not treatment with chemotherapy. Not known, NK. DI, disomic. Poly (H), high-level polysomy. Poly (L), low-level polysomy. AMP, gene amplification.

We next performed a more detailed analysis of the common subtypes of osteosarcoma, excluding surface, low-grade central, and secondary osteosarcomas (radiation-induced and Pagetic sarcomas) 2. This group included 232 tumor samples from 184 patients with central high-grade osteosarcomas, comprising 75 “good responders” and 109 “poor responders”. The clinical characteristics of these cases are summarized in Table1 and Table S1. The median age at diagnosis of this group was 16 years (range 4–64 years), with a 1.83:1 male to female ratio. Nineteen patients had tumor samples with *FGFR1* gene amplification. Of these 19 patients, 13 were male and 6 female. The median age at presentation in this group was 17 years (range 8–56).

The histological subtypes of the 184 patients with common forms of osteosarcomas are shown in Table2. The subtype distribution in the total cohort are similar to that described in the literature 2. However, *FGFR1* amplification was found to be present disproportionately in the more rare histological variants of osteosarcoma (Table2) (*P* < 0.002). The femur was the most common site for tumors, irrespective of whether they harbored *FGFR1* amplification, and all *FGFR1* amplified tumors arose in the extremities. Staging data were available for 133 patients of whom 26 (19.7%) had metastatic disease at diagnosis. Although patients with tumors harboring *FGFR1* amplification had a higher incidence of metastases (24% vs. 18%), this was not significantly different from those that did not have *FGFR1* amplification (*P* = 0.553).

**Figure 2:**
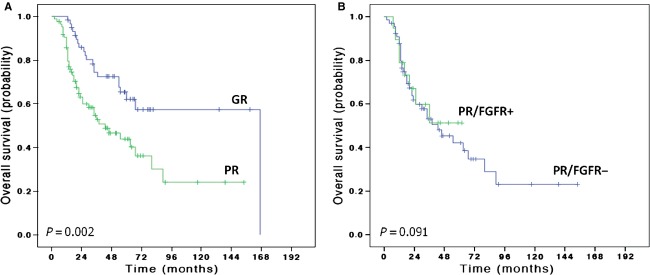
Kaplan–Meier overall survival (OS) curves of 144 osteosarcoma patients treated with neo-adjuvant chemotherapy according to response to chemotherapy (A). GR, good response; PR, poor response. Kaplan–Meier OS curves of patients with a poor response to chemotherapy according FGFR status (*FGFR1*+ = *FGFR1* amplification) (B).

Clinical outcome data were available for 144 of 176 patients with extremity tumors including all 19 patients with *FGFR1* amplification. Patients with a poor response to chemotherapy had an inferior outcome with a median OS of 43 months (95% CI 21.9–64) compared to patients whose tumors showed a good response where the median survival has not been reached after a median follow-up of 33 months (*P* < 0.001) (Fig.2). Patients with *FGFR1* amplification had an equivalent outcome to other patients with a poor response (*P* = 0.091). There was no difference in outcome of patients with polysomy CEN8 versus nonpolysomy or between those with >6 copies and <6 copies (Table S2, *P* = 0.2 and *P* = 0.39, respectively).

Only 30% of the bone osteosarcomas were diploid for CEN8 (Table4). The details of these findings in relation to the morphological subtype are found in Table4. *FGFR1* amplification was detected in two of 13 soft tissue osteosarcomas (Table1).

**Table 4:** Bone osteosarcoma cohort (275 patients) by subtype correlated with copy number status.

Histological subtype (*n*)	Diploid	Poly (H)	Poly (L)	*FGFR1* Amp	*FGFR1* AMP% by subtype
Osteoblastic (127)	34	50	36	7	5.5
Chondroblastic (49)	15	20	9	5	10.2
Fibroblastic/Pleomorphic (36)	5	15	7	9	25
Telangiectatic (20)	6	8	4	2	10
Unusual subtypes (10)	6	2	2	0	–
Low-grade central (7)	5	2	0	0	–
Surface (26)	14	5	6	1	6.8
Total (275)	85 (30.9%)	102 (37.1%)	64 (23.3%)	24 (8.7%)	

Poly (H), high-level polysomy; Poly (L), low-level polysomy; AMP, gene amplification.

## Discussion

We report the occurrence of *FGFR1* gene amplification in osteosarcoma. We found that this genetic alteration is detected in ∼10% of all bone osteosarcomas by interphase FISH. Within our cohort of 184 patients with central high-grade osteosarcomas who received neo-adjuvant chemotherapy, *FGFR1* gene amplification was exclusively detected among the “poor responder” group and represented 17.4% of patients in this group. Because of the relatively small number of cases with *FGFR1* amplification in this study, it is not possible to determine whether the survival of this patient group is significantly different to the group of “poor responders” as a whole, and whether *FGFR1* amplification in osteosarcoma contributes to the risk of metastatic disease. The cohort included patients with metastatic disease at diagnosis which may confound the outcome analysis because a higher percentage of patients with a poor response had documented metastases at presentation (22% vs. 15%), this difference was not significant (*P* = 0.261). Nevertheless, *FGFR1* gene amplification identifies a subgroup of patients who could potentially benefit from treatment with inhibitors to FGFR1.

The other significant finding of this study was that the presence of *FGFR1* amplification occurs disproportionately in the less common histological subtypes, with the highest percentage (25%) of *FGFR1* amplified cases occurring in the fibroblastic/pleomorphic subtype 2. Importantly, the spectrum and incidence of osteosarcoma subtypes in our tumor set is similar to that in the published literature, and therefore our data are not biased toward a particular histological variant 2.

*FGFR1* gene amplification was reported firstly as a potential therapeutic target in breast cancer 16–18. Amplification of the gene encoding this receptor was also detected in a number of other cancers including lung (10–22%), ovarian, and head and neck carcinomas 19–24. Similar to our finding in osteosarcoma, *FGFR1* amplification is associated with poor clinical outcome in a number of these tumor types. In particular, it was found to be the strongest independent predictor of poor outcome in estrogen receptor-positive breast cancer 16,25. Furthermore, *FGFR1* amplification in ovarian serous carcinoma is associated with increased angiogenesis, metastatic disease, and overall poor survival 24. This is the first study, however, to demonstrate specifically an association of *FGFR1* amplification with lack of response to chemotherapy. Further investigation of a role for *FGFR1* amplification in predicting chemoresistance and a potential mechanism for this is, therefore, warranted.

It is noteworthy that much of our understanding of FGF signaling in skeletal development has been obtained through study of germline alterations in the various FGFRs, which give rise to skeletal dysplasias. However, there is no evidence that such mutations are associated with the development of skeletal cancer 26.

The study by Guagnano et al. reported that the one osteosarcoma cell line which responded to anti-FGFR treatment in their in vitro study had *FGFR* amplification detected by qPCR. On analyzing the same cell line by FISH in our laboratory, we confirmed the presence of amplification by detecting 4:1 *FGFR1/*CEN8 ratio.

The difficulty in defining gene copy number as a predictive biomarker for stratification of patients for targeted therapy is well recognized even with established actionable targets, such as *HER2* in breast cancer. Here, even after many years of use within clinical practice there is still no consensus defining which level of copy number gain predicts the response to inhibitors 27–29. Well-designed clinical studies correlated with gene copy number for which there are strict and reproducible scoring criteria should provide an evidence base for patient stratification.

In the study by Guagnano et al., it was also demonstrated that the FGFR-kinase inhibitor exerted no effect on 54% (*n*-20) of cell lines harboring *FGFR1* amplification. In some of these tumors, additional alterations were detected including mutations in *KRAS (n = 4)*, and *BRAF,* and amplification of *HER2*, which may contribute to the resistance to the FGFR-kinase inhibitor 13. These findings emphasize that, as technology develops, patients may benefit from more global genomic analysis of tumor samples prior to treating with targeted compounds.

This is the first study to show a genetic alteration in osteosarcomas that is associated with a poor response to neo-adjuvant chemotherapy. Although prospective validation of results is warranted, a number of FGFR1 inhibitors are currently in clinical development, and phase II biomarker-driven studies are already underway in several solid tumors http://clinicaltrials.gov/ct2/show/NCT01761747?term=NCT01761747&rank=1.

This study provides a rationale for inclusion of osteosarcoma patients in such studies. The challenge will be how best to determine selection criteria for their inclusion, and how to encourage pharmaceutical companies to undertake clinical trials involving this rare cancer.

## Supporting Information

**Table S1.** Clinical characteristics of 184 patients with osteosarcoma according to *FGFR1* status. **Table S2.** Clinical characteristics and outcome analysis of 144 patients with extremity osteosarcoma.
