# Supplementary material for: Fibroblastic growth factor receptor 1 amplification in osteosarcoma is associated with poor response to neo-adjuvant chemotherapy
Source: Cancer Med. 2014 May 27;3(4):980–7. doi: 10.1002/cam4.268 (PMC4303166; doi:10.1002/cam4.268)
Supplement: Supplementary file 1 [file cam40003-0980-sd1.docx]

**Table S01.** Clinical characteristics of 184 patients with osteosarcoma according to *FGFR1* status

|  |  | **FGFR negative** | | **FGFR positive** | |
| --- | --- | --- | --- | --- | --- |
|  |  |  |  |  |  |
| Total |  | 165 | | 19* | |
|  |  |  |  |  |  |
| Median Age, yrs (range) | | 16 (4-64) | | 17 (8-56) | |
| Sex, M:F |  | 106:59 | | 13:6 | |
|  |  |  |  |  |  |
| Primary Site | | No. | % | No. | % |
|  | Extremity |  |  |  |  |
|  | Femur | 87 | (52) | 9 | (47) |
|  | Tibia | 42 | (25) | 2 | (11) |
|  | Fibula | 9 | (5) | 2 | (11) |
|  | Humerus | 13 | (8) | 4 | (21) |
|  | radius/ulna | 1 | (1) | 2 | (11) |
|  |  |  |  |  |  |
|  | Pelvis/trunk |  |  |  |  |
|  | pelvis | 7 | (4) | 0 | (0) |
|  | scapula | 3 | (2) | 0 | (0) |
|  | rib | 1 | (1) | 0 | (0) |
|  |  |  |  |  |  |
|  | Small bones | 2 | (1) | 0 | (0) |
|  |  |  |  |  |  |
| Metastatic at diagnosis | | No. | % | No. | % |
|  |  |  |  |  |  |
| No |  | 96 | (77) | 11 | (64) |
|  |  |  |  |  |  |
| Yes |  | 22 | (18) | 4 | (24) |
|  |  |  |  |  |  |
|  | Lung | 14 | (11) | 2 | (12) |
|  | Bone | 6 | (5) | 1 | (6) |
|  | Lung and bone | 2 | (1) | 1 | (6) |
|  |  |  |  |  |  |
| Equivocal |  | 7 | (6) | 2 | (12) |
|  |  |  |  |  |  |
| (Not known) | | 40 | 24 | 2 | 10 |

*Distribution of positive cases as described in the methods section: 7/19 in which ≥10% of cells showed *FGFR1*/CEN8 ratio>2; 6/19 in which there were clusters of *FGFR1* signals per cell; 6/19 in which there were >15 copies of *FGFR1* per cell.

**Table S02.** Clinical characteristics and outcome analysis of 144 patients with extremity osteosarcoma

|  | | | Good response  (n=59) | | | Poor response  (n=85) | | | | | |
| --- | --- | --- | --- | --- | --- | --- | --- | --- | --- | --- | --- |
|  |  | |  |  | | FGFR negative  (n=66) | | | FGFR positive  (n=19) | | |
|  |  | |  |  | |  | |  |  | |  |
| Median Age, yrs (range) | | | 16 (4-64) | | | 17 (6-58) | | | 17 (8-56) | | |
| Sex, M:F |  | | 34:25 (1.4:1) | | | 46:20 (2.3:1) | | | 13:6 (2.1:1) | | |
|  |  | |  |  | |  | |  |  | |  |
| Primary Site, No (%) | | | | | | | | | | | |
|  | | Extremity |  | |  |  |  | |  |  | |
|  | | Femur | 33 | | (56) | 39 | (60) | | 9 | (47.4) | |
|  | | Tibia | 18 | | (31) | 17 | (26) | | 2 | (10.5) | |
|  | | Fibula | 2 | | (3) | 5 | (7) | | 2 | (10.5) | |
|  | | Humerus | 6 | | (10) | 4 | (6) | | 4 | (21.1) | |
|  | | radius/ulna | 0 | | (0) | 1 | (1) | | 2 | (10.5) | |
|  | |  |  | |  |  |  | |  |  | |
| Metastatic at diagnosis, No. (%) | | | | | | | | | | | |
| No | |  | 43 | | (81) | 41 | (69) | | 11 | (64) | |
|  | |  |  | |  |  |  | |  |  | |
| Yes | |  | 8 | | (15) | 13 | (22) | | 4 | (24) | |
|  | | Lung | 7 | | (13) | 6 | (10) | | 2 | (12) | |
|  | | Bone | 1 | | (2) | 5 | (8) | | 1 | (6) | |
|  | | Lung and bone | 0 | | (0) | 2 | (3) | | 1 | (6) | |
|  | |  |  | |  |  |  | |  |  | |
| Equivocal | |  | 2 | | (4) | 5 | (8) | | 2 | (12) | |
|  | |  |  | |  |  |  | |  |  | |
| (Not known) | | | 6 | | (10) | 7 | (11) | | 2 | (11) | |
|  | | |  | |  |  |  | |  |  | |
| Outcome | | | | | | | | | | | |
|  |  | |  | |  |  |  | |  |  | |
| Alive |  | | 38 | | (65) | 31 | (47) | | 11 | (58) | |
| Dead |  | | 21 | | (35) | 35 | (53) | | 8 | (42) | |
